# A Multi-Method Examination of Ageism in Children Before and During the Pandemic

**DOI:** 10.1093/geroni/igab046.2267

**Published:** 2021-12-17

**Authors:** Jennifer Bellingtier, Jenny Jaquet (née Bauer), Lena-Emilia Schenker

**Affiliations:** Friedrich Schiller University Jena, Jena, Thuringen, Germany

## Abstract

The pandemic has made age more salient. Access to vaccines, mandates to wear masks, and recommendations for contact restrictions have all varied by age. Developmental intergroup theory proposes that greater salience of a feature can lead to greater stereotyping and prejudice. We investigated this with a multi-method assessment of ageism in children (N = 57, ages 4-8), where data collection occurred both before and during the pandemic. In simulated behavioral measures, children preferred to sit closer to younger adults (mean distance = 1.8 seats) versus older adults (mean distance = 2.8 seats), and, for a simulated treasure hunt, they chose 3.36 younger, versus 1.63 older, teammates. Explicit (picture ratings) and implicit (IAT) ratings also significantly favored younger adults. These preferences were not exacerbated by the pandemic. Although ageism is present at young ages, we found no evidence that this has thus far worsened in the pandemic.

